# Single-Compartment Dose Prescriptions for Ablative ^90^Y-Radioembolization Segmentectomy

**DOI:** 10.3390/life13061238

**Published:** 2023-05-24

**Authors:** Srinivas Cheenu Kappadath, Benjamin P. Lopez

**Affiliations:** Department of Imaging Physics, UT MD Anderson Cancer Center, 1155 Pressler St., Unit 1352, Houston, TX 77030, USA

**Keywords:** radioembolization, yttrium-90, dosimetry, segmentectomy, margin

## Abstract

Background: Yttrium-90 (^90^Y) radioembolization is increasingly being utilized with curative intent. While single-compartment doses with respect to the perfused volume for the complete pathologic necrosis (CPN) of tumors have been reported, the actual doses delivered to the tumor and at-risk margins that leads to CPN have hitherto not been estimated. We present an ablative dosimetry model that calculates the dose distribution for tumors and at-risk margins based on numerical mm-scale dose modeling and the available clinical CPN evidence and report on the necessary dose metrics needed to achieve CPN following ^90^Y-radioembolization. Methods: Three-dimensional (3D) activity distributions (MBq/voxel) simulating spherical tumors were modeled with a 121 × 121 × 121 mm^3^ soft tissue volume (1 mm^3^ voxels). Then, 3D dose distributions (Gy/voxel) were estimated by convolving 3D activity distributions with a ^90^Y 3D dose kernel (Gy/MBq) sized 61 × 61 × 61 mm^3^ (1 mm^3^ voxels). Based on the published data on single-compartment segmental doses for the resected liver samples of HCC tumors showing CPN after radiation segmentectomy, the nominal voxel-based mean tumor dose ($D_{mean}^{CPN}$), point dose at tumor rim ($D_{rim}^{CPN}$), and point dose 2 mm beyond the tumor boundary ($D_{2mm}^{CPN}$), which are necessary to achieve CPN, were calculated. The single-compartment dose prescriptions to required achieve CPN were then analytically modeled for more general cases of tumors with diameters $d_{t}$ = 2, 3, 4, 5, 6, and 7 cm and with tumor-to-normal-liver uptake ratios T:N = 1:1, 2:1, 3:1, 4:1, and 5:1. Results: The nominal case defined to estimate the doses needed for CPN, based on the previously published clinical data, was a single hyperperfused tumor with a diameter of 2.5 cm and T:N = 3:1, treated with a single-compartment segmental dose of 400 Gy. The voxel-level doses necessary to achieve CPN were 1053 Gy for the mean tumor dose, 860 Gy for the point dose at the tumor boundary, and 561 Gy for the point dose at 2 mm beyond the tumor edge. The single-compartment segmental doses necessary to satisfy the criteria for CPN in terms of the mean tumor dose, point dose at the tumor boundary, and the point dose at 2 mm beyond the tumor edge were tabulated for a range of tumor diameters and tumor-to-normal-liver uptake ratios. Conclusions: The analytical functions that describe the relevant dose metrics for CPN and, more importantly, the single-compartment dose prescriptions for the perfused volume needed to achieve CPN are reported for a large range of conditions in terms of tumor diameters (1–7 cm) and T:N uptake ratios (2:1–5:1).

## 1. Introduction

Radioembolization is a form of brachytherapy where radioactive microspheres, most commonly containing yttrium-90 (^90^Y), are administered trans-arterially into selectable vasculature(s) supplying tumor(s) within the liver [1,2]. After infusion, the microspheres (nominally 30 μm in diameter with a range of 20–60 μm) travel passively via the arterial blood flow into the vascular network and become trapped in the capillaries due to their size. The emission of beta particles during ^90^Y decay (energetic electrons with a maximum energy of 2.23 MeV and mean energy of 0.938 MeV) from the trapped microspheres is the actuator of therapeutic radiation. The concentration of ^90^Y-microspheres implanted in the tissue is proportional, among other factors, to the vascularity of the perfused tissue; consequently, hyperperfused tissues such as tumors receive a high load of ^90^Y-microspheres and therefore receive a higher radiation dose.

**Figure 1:**
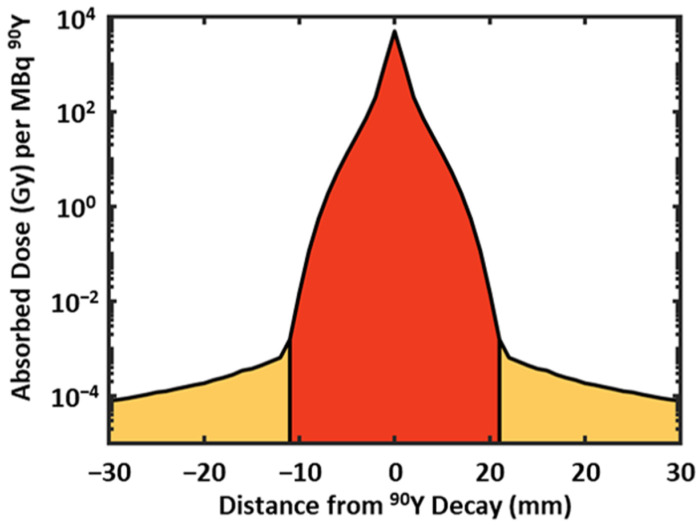
Simulated ^90^Y absorbed radiation dose kernel for 1 mm^3^ voxels. Central ±11 mm region (red) is dominated by beta particle dose while peripheral regions (orange) are dominated by bremsstrahlung photon dose (photon contributions <<10^−6^ of overall dose).

^90^Y is a pure beta emitter with a mean beta range of 2–4 mm, with >99.9% of the overall absorbed dose deposited within 11 mm (Figure 1) [3]. There exists a low likelihood of the generation of bremsstrahlung photons that can lead to dose deposition beyond the beta particle range; however, its contribution to the overall tissue dose is extremely low. The beta particle range has negative and positive implications for tumor and normal tissue dose deposition. A negative consequence of the beta particle range is the potential for the under-dosing of tumor margins, even in ideal cases of tumors that are uniformly perfused with microspheres. The resultant mean tumor dose is therefore lower than initially planned, especially in smaller tumors (diameters < 2 cm). Conversely, the beta particle range has the potential benefit of irradiating volumes with a low microsphere uptake, as in the case of tissues just beyond the tumor margins or hypo-perfused regions within tumors.

Curative-intent tumor ablation is now largely supported across guidelines for the treatment of hepatic malignancies, such as early stage hepatocellular carcinoma (HCC) [4,5,6]. While ablation is conventionally used to describe thermal modalities, the definition, in principle, may apply to any in situ therapy, including radiation, generating controlled and irreversible tissue destruction. Consequently, radioembolization offers ablative capabilities when tumors are treated with high radiation dose levels. This is the definition of ablative radioembolization. Ablative radioembolization, best represented by radiation segmentectomy, has been reported to show favorable outcomes in the treatment of HCC with respect to both safety and efficacy [7,8,9,10,11].

Radioembolization dosimetry, initially driven by empiric models for safety, has progressed from single-compartment uniform-uptake (henceforth referred to as the *Standard*) dosimetry to multi-compartment *Partition* (uniform-uptake within separate tumor and normal liver compartments) dosimetry and then to *Voxel* dosimetry for the estimation of both tumor and normal liver doses that can guide treatment intent [12,13,14,15]. For example, there is a growing consensus derived from lobar and whole-liver treatments with ^90^Y-glass radioembolization that HCC tumor mean doses of around 200–350 Gy (based on multi-compartment *Partition* dosimetry) are needed for imaging-based responses, such as RECIST or mRECIST [13,16,17,18].

Furthermore, retrospective analyses of patients who were first treated with ablative radioembolization and then underwent liver resection or transplantation after ^90^Y-glass radioembolization showed a strong correspondence between radiation segmentectomy and complete pathologic necrosis (CPN). In a 2014 retrospective study of 102 HCC patients treated with a median treatment volume of 165 mL (range 108–240 mL) and median tumor diameter of 2.6 cm (range 2.1–3.6 cm), 33 patients underwent transplantation following radiation segmentectomy [19]. Among these 33, 14 of the 17 (86%) patients who received *Standard* dosimetry segmental doses >190 Gy with respect to the perfused volume exhibited CPN. In a 2020 retrospective analysis of 45 HCC patients (2.5 cm median tumor diameters) with resected liver samples, 100% CPN was achieved when the *Standard* dosimetry segmental doses with respect to the perfused volume were >400 Gy [20].

As mentioned, *Standard* dosimetry can estimate only the mean dose with respect to the entire treatment volume; therefore, the actual doses delivered to tumors in these studies, albeit unknown, were likely substantially larger than the reported mean segmental doses due to the preferential deposition of microspheres in hypervascular tumors relative to normal tissues within the segmental volume. HCC tumors are known to be hypervascular with preferential uptake relative to perfused normal liver tissues, with reported HCC tumor-to-normal-liver uptake ratios (T:N) of 2.3 to 3.6 [11,17,21,22].

Recent studies using radiofrequency ablation, trans-arterial chemoembolization (which also takes advantage of the arterial vascularization of the tumors), and resection have demonstrated improved outcomes when the tumor margins (+3 to +5 mm) were appropriately treated, advocating for the treatment of tumor margins during ablative radioembolization [23,24]. Because microsatellites are commonly present around tumors, the high segmental doses (>400 Gy) that lead to complete pathologic necrosis may, in part, be due to the adequate doses for at-risk tumor margins that accompany high-activity administrations. Therefore, to improve dosimetry treatment planning in lobar and whole-liver approaches, there is a need to better understand the *Voxel* doses delivered to the tumor and at-risk margins in radiation segmentectomy with *Standard* dosimetry that have resulted in a complete pathological response.

The notion of prescribing a dose to tumor margins has parallels in radiotherapy planning, where doses are prescribed to gross tumor volumes (GTV) and clinical tumor volumes (CTV). By definition, the GTV describes the extent of the primary tumor, while the CTV encompasses the GTV and captures the extent of microscopic tumor spread [25]. Although the GTV is easy to define conceptually, in practice, the edges of the GTV are not often clearly delineated on imaging. The delineation of CTV is defined as the GTV with a margin for sub-clinical disease spread not characterized through imaging; the extent of microscopic disease spread is typically based on a historical series rather than the extent of GTV in an individual patient. Studies have suggested a 5 mm margin as appropriate for HCC ablation margins [23]. Therefore a 2 mm expansion of GTV may be a reasonable description of the CTV for radioembolization.

There were two related objectives of this work. The first objective was to use high-resolution computational tumor models accounting for beta particle mobility and transport to estimate the nominal absorbed *Voxel* doses delivered to the simulated total tumor volume, tumor edge, and tumor +2 mm margin that lead to complete pathological necrosis in resected liver samples after ablative radiation segmentectomy [9,20]. The second objective was to then characterize the prescribed dose for the perfused treatment volume in terms of *Standard* dosimetry, as a function of the tumor size and uptake needed to achieve ablative radiation segmentectomy based on the dose according to the total tumor volume, the tumor’s edge, and the tumor’s +2 mm margin.

This work is expected to generate important information for radioembolization practitioners on the actual tumor and margin doses that lead to the achievement of complete pathologic necrosis and, more importantly, recommendations for radiation absorbed dose modification schemas depending on the patient-specific tumor characteristics of size and uptake yet based on the commonly used *Standard* dosimetry model, which will lead to a higher probability of complete pathologic necrosis in routine clinical practice.

## 2. Materials and Methods

### 2.1. Mathematical Schema Relating to Partition and Voxel Dosimetry

A total of 28 spherical tumors were simulated in tissues with various ^90^Y-activity distributions that consisted of 7 different tumor diameters ($d_{t}\;$= 1, 2, 3, 4, 5, 6, and 7 cm) and 4 distinct normal-to-tumor voxel activity concentration ratios ($A_{t}^{n}$ = 0, 0.1, 0.2, 0.3) with a 121 × 121 × 121 mm^3^ soft tissue volume (1 mm^3^ voxels, 1.04 g/cm^3^ density). These tumor conditions were selected to span the expected clinical range of tumor sizes and uptakes. For the purpose of developing our mathematical schema, tumor voxels were uniformly filled with activity, such that the total tumor activity resulted in a *Partition* dose of $D_{partition}$ = 1000 Gy (assuming 49.67 Gy·g/MBq). Once filled with the appropriate tumor and normal tissue ^90^Y activities (MBq), simulated activity distributions were convolved using a 3D isotropic ^90^Y dose activity kernel (Gy/MBq) to obtain absorbed voxel dose distributions (Gy). The ^90^Y dose activity kernel (61 × 61 × 61 mm^3^, 1 mm^3^ voxels) was calculated using the EGSnrc user code DOSXYZnrc to track all the emitted beta particle and bremsstrahlung photon energy depositions [26,27]. All calculations related to the activity modeling and dose convolutions were performed in MATLAB (MathWorks, Natick, MA, USA).

Two dose metrics were calculated: (1) $R_{mean}$, defined as the ratio of the *Voxel*-based mean dose to the total tumor volume ($D_{mean}$) to the *Partition*-based tumor mean dose ($D_{partition}$), and (2) $R_{edge}$, defined as the ratio of the *Voxel*-based dose at and adjacent to the tumor’s margin ($D_{edge}$) to the $D_{partition}$. $R_{mean}$ was modeled as an analytic function of $A_{t}^{n}$ and *d_t_*, while $R_{edge}$ was modeled as an analytic function of $A_{t}^{n}$ and x, where x is the distance from the tumor edge.

Both $R_{mean}\left(d_{t},A_{t}^{n}\right)$ and $R_{edge}\left(A_{t}^{n},x\right)$ were modeled in MATLAB using the nonlinear least squares fitting method with the Levenberg–Marquardt algorithm. Different analytical models were investigated for each dose metric with varying complexity (e.g., linear vs. quadratic vs. exponential) and varying number of fit coefficients. The final analytical models were heuristically selected to maximize the goodness-of-fit *R*^2^ value while minimizing the model complexity and number of coefficients.

### 2.2. Modeling Standard, Partition, and Voxel Dosimetry for CPN

Based on the published data on resected liver samples of HCC tumors after ablative radiation segmentectomy [19,20], the nominal *Standard* segmental dose necessary to achieve CPN was defined as $D_{std}^{CPN}$ = 400 Gy for a $d_{t}$ = 2.5 cm tumor. Assuming a typical 3:1 tumor-to-normal-liver uptake ratio ($A_{t}^{n}=0.33$), this segmental dose would correspond to a *Partition* tumor dose of $D_{partition}^{CPN}$ = 1200 Gy. These nominal tumor conditions were input into the analytical functions developed to describe $R_{mean}$($A_{t}^{n},\;d_{t})$ and $R_{edge}\left(A_{t}^{n},\;x\right)\;$ in order to estimate the expected *Voxel* mean tumor dose ($D_{mean}^{CPN}$), point dose at the tumor rim ($D_{rim}^{CPN}$), and point dose 2 mm beyond the tumor boundary ($D_{2mm}^{CPN}$) and thus achieve CPN.

Assuming all 3 $D_{mean}^{CPN}$, $D_{rim}^{CPN}$, and $D_{2mm}^{CPN}$ *Voxel* doses must be satisfied to achieve CPN, we report the *Standard* segmental and *Partition* tumor dose prescriptions necessary to achieve CPN for tumors with diameters $d_{t}$ = 2, 3, 4, 5, 6, and 7 cm and with tumor-to-normal-liver uptake ratios T:N = 1:1, 2:1, 3:1, 4:1, and 5:1.

## 3. Results

### 3.1. Mathematical Schema Relating to Partition and Voxel Dosimetry

$R_{mean}$, the ratio of the *Voxel*-based mean dose to the total tumor volume ($D_{mean}$) to the *Partition*-based tumor mean dose ($D_{partition}$), modeled as an analytic function of $A_{t}^{n}$ and $d_{t}$, was found to be best described in the case of tumors with ≥1 cm diameter, as follows:

(1) $$R_{mean}\left(d_{t},A_{t}^{n}\right)=1-\left(\frac{c_{1}}{d_{t}}\right)\left(1-A_{t}^{n}\right)=1-\left(\frac{0.46}{d_{t}}\right)\left(1-A_{t}^{n}\right)$$

where $d_{t}$ is the tumor diameter in cm and $A_{t}^{n}$ is the normal-to-tumor-tissue activity concentration ratio ($0\leq A_{t}^{n}\leq 1$). This model had a single free coefficient $c_{1}$ = 0.46 (95% confidence interval = 0.44–0.47) and a resulting *R*^2^ = 0.987.

$R_{edge}$, the ratio of the *Voxel*-based dose at and adjacent to the tumor’s margin ($D_{edge}$) to the $D_{partition}$, modeled as an analytic function of $A_{t}^{n}$ and x in mm, was found to be best described in the case of tumors with ≥2 cm diameter, as follows:

(2) $$R_{edge}\left(A_{t}^{n},x\right)=\left[\frac{1}{c_{2}\cdot e^{c_{3}x}+1}\right]+\left[\frac{A_{t}^{n}}{c_{2}\cdot e^{-c_{3}x}+1}\right]=\left[\frac{1}{1.3\cdot e^{0.8x}+1}\right]+\left[\frac{A_{t}^{n}}{1.3\cdot e^{-0.8x}+1}\right]$$

where x= −1 mm is the last tumor voxel at the boundary, x= 0 mm is first normal tissue voxel at the boundary, and +x mm are normal tissue voxels away from the tumor boundary. This model had two free coefficients of $c_{2}$ = 1.3 (95% confidence interval of 1.31–1.33) and $c_{3}$ = 0.8 (95% confidence interval 0.77–0.80) and a resulting *R^2^* = 0.999. For all tumors with $d_{t}$ > 2 cm, the dose profiles for the tumor margin (±15 mm centered at the tumor margin) varied by less than 1% for all tumors with the same background activity ratio ($A_{t}^{n}$).

The resulting relationships between the *Partition* tumor ($D_{partition}$) and *Standard* segmental ($D_{std}$) doses and the *Voxel* mean tumor doses ($D_{mean}$), the point dose at the tumor rim ($D_{rim}$), and the point dose 2 mm beyond tumor boundary ($D_{2mm}$) can be rewritten based on Equations (1) and (2) as a function of the tumor diameter ($d_{t}$, cm) and tumor-to-normal-tissue uptake ratio (T:N) as follows:

(3) $$D_{mean}\left(d_{t},T:N\right)=D_{partition}\times \left[1-\left(\frac{0.46}{d_{t}}\right)\left(1-\frac{1}{T:N}\right)\right],$$

(4) $$D_{rim}\left(T:N\right)=D_{partition}\times \left[\left(\frac{1}{1.3\cdot e^{-0.8}+1}\right)+\left(\frac{1}{T:N}\right)\left(\frac{1}{1.3\cdot e^{0.8}+1}\right)\right],$$

(5) $$D_{2mm}\left(T:N\right)=D_{partition}\times \left[\left(\frac{1}{1.3\cdot e^{0.8}+1}\right)+\left(\frac{1}{T:N}\right)\left(\frac{1}{1.3\cdot e^{-0.8}+1}\right)\right],$$

and

(6) $$D_{partition}=D_{std}\times \left(T:N\right).$$

### 3.2. Tumor Mean and Margin Doses for CPN in the Nominal Case

The nominal case defined to estimate doses needed for CPN, based on the previously published clinical data [19,20], was a single hyperperfused tumor of $d_{t}$ = 2.5 cm and $A_{t}^{n}\;$= 0.33 (i.e., T:N = 3:1) treated with the *Standard* dosimetry dose for the perfused volume of $D_{std}^{CPN}$ = 400 Gy. These conditions correspond to normal liver and tumor voxel activity concentrations of 25.1 MBq/mL and 8.4 MBq/mL, respectively. Using Equations (3)–(6), the *Voxel* doses necessary to achieve CPN are $D_{mean}^{CPN}$ = 1053 Gy for the mean tumor dose, $D_{rim}^{CPN}$ = 860 Gy for the dose at the tumor boundary, and $D_{2mm}^{CPN}$ = 561 Gy for the dose 2 mm beyond the tumor edge.

### 3.3. Prescribing Tumor Mean Doses for CPN in a General Case

The *Standard* segmental doses calculated using Equation (3) that are necessary to satisfy $D_{mean}$ ≥ $D_{mean}^{CPN}$ = 1053 Gy for a range of tumor diameters and tumor-to-normal-liver uptake ratios are shown in Table 1. The change in the tumor-to-normal-tissue ratio had a greater effect than the tumor size on the prescribed single-compartment dose for the perfused volume. For instance, the prescribed single-compartment dose for a $d_{t}$ = 3 cm tumor decreased from 391 Gy to 240 Gy when T:N  increased from 3:1 to 5:1, while the prescribed single-compartment dose for a tumor with 3:1 T:N only decreased from 391 Gy to 370 Gy when $d_{t}$ increased from 3 cm to 6 cm (an 800% increase in tumor volume).

Generally, the prescribed single-compartment *Segmental* dose for the perfused volume necessary to achieve CPN is predicted to be ≤400 Gy for tumors of 2 cm in diameter or more and uptake ratios of 3:1 or more. For instance, a 3 cm diameter tumor would receive the same mean tumor dose as the nominal tumor condition with CPN, with prescribed *Segmental* doses of only 297 Gy and 240 Gy, respectively, if the estimated T:N uptake ratios were 4:1 and 5:1. Conversely, the prescribed single-compartment dose for the perfused volume would need to be >400 Gy, or even up to 600 Gy, for tumors of all sizes with a T:N less than 3:1 in order to achieve the same mean tumor dose for CPN.

### 3.4. Prescribing Tumor Margin Doses for CPN in a General Case

The *Standard* segmental doses calculated using Equations (4) and (5) that are necessary to satisfy $D_{rim}$ ≥ $D_{rim}^{CPN}$ = 860 Gy and or $D_{2mm}$ ≥ $D_{2mm}^{CPN}$ = 561 Gy for a range of tumor-to-normal-liver uptake ratios are shown in Table 2. The dose profiles of the tumor margins were largely independent of the tumor size (when $d_{t}$ > 2 cm) and were therefore modeled only as a function of the T:N ratio. As expected, the point dose at 2 mm beyond the tumor margin was lower than those at the tumor margin for a given value of the prescribed single-compartment dose for the perfused volume.

### 3.5. Prescribing Segmental Doses for CPN in a General Case

The necessary *Standard* segmental doses that can satisfy all three criteria for CPN, namely, $D_{mean}$ ≥ $D_{mean}^{CPN}$ = 1053 Gy, $D_{rim}$ ≥ $D_{rim}^{CPN}$ = 860 Gy, and $D_{2mm}$ ≥ $D_{2mm}^{CPN}$ = 561 Gy, are shown in Table 3. The metrics $D_{mean}^{CPN}$ and $D_{2mm}^{CPN}$ were both observed to drive the combined dose requirement. On average, tumors $d_{t}\geq$ 3 cm and T:N≥ 3 were driven by $D_{2mm}^{CPN}$, while the complement conditions were driven by $D_{mean}^{CPN}$.

## 4. Discussion

Clinical data have been reported on tumor CPN with single-compartment dosimetry with respect to the perfused volume, yet the actual doses delivered to the tumor and the at-risk margins that lead to CPN have hitherto not been estimated. This work is the first to perform calculations of the dose distribution for tumors and at-risk margins based on numerical mm-scale dose modeling and the available clinical CPN evidence and report on the necessary dose metrics needed to achieve CPN following ^90^Y-radioembolization.

In addition to the mean tumor dose for CPN (1053 Gy $D_{mean}^{CPN}$), this paper describes the point dose for the tumor rim that ensures complete tumor dose coverage (860 Gy $D_{rim}^{CPN}$) and the point dose 2 mm outside the visible margin that accounts for the dose for microscopic disease (561 Gy $D_{2mm}^{CPN}$), all representing conditions necessary to achieve CPN after ^90^Y-radioembolization. The behavior of $D_{mean}$, $D_{rim}$, and $D_{2mm}$ were characterized as analytical functions of the patient-specific tumor diameters and tumor-to-normal-liver uptake ratios. Furthermore, the prescribed doses, based on the commonly use *Standard* dosimetry model, that satisfy $D_{mean}$ ≥ $D_{mean}^{CPN}$ = 1053 Gy, $D_{rim}$ ≥ $D_{rim}^{CPN}$ = 860 Gy, and $D_{2mm}$ ≥ $D_{2mm}^{CPN}$ = 561 Gy, were tabulated for a range of tumor diameters and tumor-to-normal-liver uptake ratios. Finally, segmental doses (in Gy) based on the *Standard* dosimetry model that simultaneously satisfy $D_{mean}$ ≥ $D_{mean}^{CPN}$, $D_{rim}$ ≥ $D_{rim}^{CPN}$, and $D_{2mm}$ ≥ $D_{2mm}^{CPN}$, leading to a higher probability of complete pathologic necrosis in routine clinical practice, were also provided for a range of tumor diameters and tumor-to-normal-liver uptake ratios.

The dosimetry model presented here should be regarded as a foundational conceptual model that could help to better rationalize the larger tumor mean doses necessary for complete pathologic necrosis relative to those needed for radiological responses. One should recall that lobar and whole-liver treatments with ^90^Y-glass radioembolization have shown HCC tumor mean doses of around 200–350 Gy (based on multi-compartment dosimetry) necessary for radiographic responses [13,16].

Furthermore, this work provides the analytical functions that describe the behavior of the relevant dose metrics and, more importantly, the single-compartment dose prescriptions for the perfused volume needed to achieve CPN doses for a large range of tumor conditions in terms of tumor diameters (1–7 cm) and T:N  uptake ratios (2:1–5:1).

The clinical workflow envisioned to plan ablative ^90^Y-radioembolization based on this work will start the with identification of the expendable volume of liver tissue containing the tumor for radioembolization treatment. Imaging will be used to assess patient-specific tumor characteristics such as size and uptake; the tumor size is usually determined using diagnostic computed tomography (CT) or angiography CT/cone-beam CT, whereas the uptake is usually determined using ^99m^Tc-MAA single photon emission computed tomography/CT (SPECT/CT). The ablative dosimetry model, introduced in this work, will then be used to quantify the necessary prescribed single-compartment dose with respect to the perfused volume, based on the patient-specific tumor characteristics of tumor size and uptake, to achieve the desired dose metrics for CPN. The largest of the prescribed doses necessary to achieve either $D_{mean}^{CPN}$, $D_{rim}^{CPN}$, or $D_{2mm}^{CPN}$ will be selected by the user for the planning of ablative ^90^Y-radioembolization (Table 3).

Two “clinical” scenarios are presented to illustrate the dosimetry concepts developed in this work for ablative radiation segmentectomy. In the following cases, the user-prescribed goal is to achieve CPN for the single tumor in the expendable perfused volume. For simplicity, lung dose considerations are ignored.

**Figure 2:**
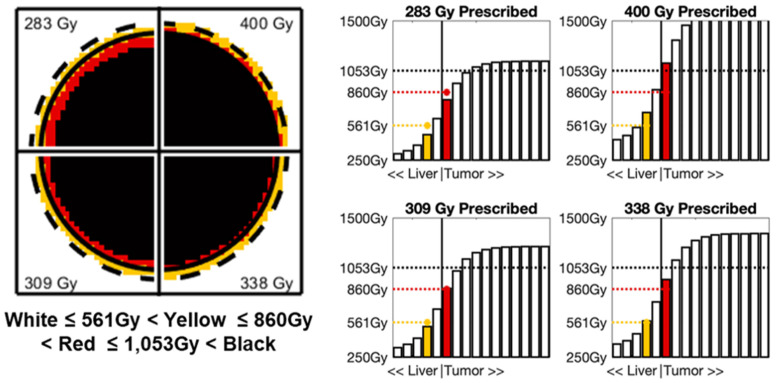
Resulting dose maps through the center of the tumor and line profiles near edge of tumor for Case 1:5 cm diameter tumor in a 200 mL segment with *T*:*N* of 5:1 at 4 different prescribed segmental doses (283 Gy, 309 Gy, 338 Gy, and 400 Gy). In dose map, solid circle represents tumor rim, dashed circle represents 2 mm margin, and voxel doses are color coded in 4 dose ranges. In line profiles, yellow bar represents 2 mm margin and desired $D_{2mm}^{CPN}$ = 561 Gy and red bar represents tumor margin and desired $D_{rim}^{CPN}$ = 860 Gy.

Case 1 entails a 5 cm diameter (65 mL) uniformly perfused tumor with a tumor-to-normal-liver uptake ratio of 4:1. Depending on the target *Voxel* dose of interest, the *Standard* single-compartment segmental dose necessary to achieve CPN will be 283 Gy (for $D_{mean}>D_{mean}^{CPN}$), 309 Gy (for $D_{rim}>D_{rim}^{CPN}$), and 338 Gy (for $D_{2mm}>D_{2mm}^{CPN}$) based on Table 2 and Table 3. Assuming a segmental volume of 200 mL, the necessary activity prescriptions will be 1.19 GBq, 1.29 GBq, and 1.42 GBq, respectively. Figure 2 shows the corresponding 2D dose maps and 1D dose profiles throughout the tumor center for each of the three activity prescriptions. When $D_{std}\;$283 Gy was prescribed, the resulting rim (787 Gy) and margin (469 Gy) point doses were below their respective CPN thresholds. When $D_{std}\;$320 Gy was prescribed, the resulting mean tumor dose (1151 Gy) was greater than the $D_{mean}^{CPN}$ but the margin point dose (513 Gy) was still below $D_{2mm}^{CPN}$. Finally, when $D_{std}\;$359 Gy was prescribed, all three dose metrics exceeded their respective CPN thresholds (1259 Gy > $D_{mean}^{CPN}$, 940 Gy > $D_{rim}^{CPN}$, and 561 Gy > $D_{2mm}^{CPN}$). Therefore, a prescribed *Standard* single-compartment segmental dose ≥ 359 Gy would maximize the probability of the tumor achieving CPN.

**Figure 3:**
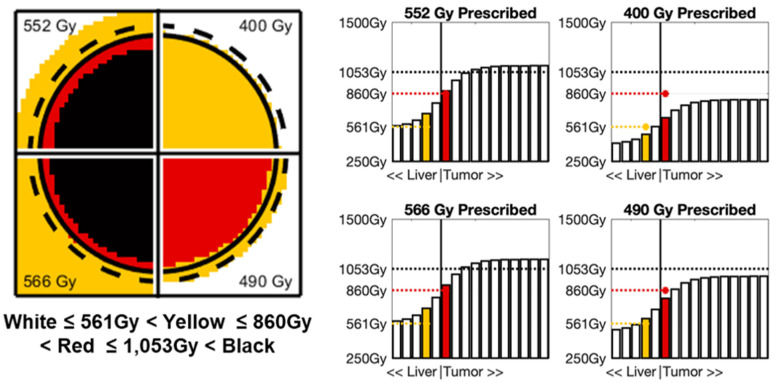
Resulting dose maps through the center of the tumor and line profiles near edge of tumor for Case 2:5 cm diameter tumor in a 200 mL segment with T:N of 2:1 at 4 different prescribed segmental doses (552 Gy, 566 Gy, 490 Gy, and 400 Gy). In line profiles, yellow bar represents 2 mm margin and desired $D_{2mm}^{CPN}$ = 561 Gy and red bar represents tumor margin and desired $D_{rim}^{CPN}$ = 860 Gy.

Case 2 (Figure 3) entails the same 5 cm diameter tumor but now with a lower uptake ratio of 2:1. The *Standard* single-compartment segmental doses necessary to achieve CPN in Case 2 are 552 Gy (for $D_{mean}>D_{mean}^{CPN}$), 566 Gy (for $D_{rim}>D_{rim}^{CPN}$), and 490 Gy (for $D_{2mm}>D_{2mm}^{CPN}$) based on Table 2 and Table 3. In Case 2, all three dose metrics exceed their respective CPN thresholds when *Standard* dosimetry of 552 Gy (2.31 GBq) is prescribed (1053 Gy > $D_{mean}^{CPN}$, 839 Gy > $D_{rim}^{CPN}$, and 638 Gy > $D_{2mm}^{CPN}$) and thus also when 566 Gy (2.37 GBq) is prescribed. However, if only 490 Gy (2.05 GBq) is prescribed, the resulting mean tumor dose (935 Gy) will not exceed $D_{mean}^{CPN}$. Therefore, a prescribed single-compartment dose ≥ 552 Gy will maximize the probability of the tumor achieving CPN. In other words, prescribing a 400 Gy *Standard* segmental dose to both tumors would be an “overkill” in Case 1 but insufficient in Case 2.

Some of the limitations of the work presented here are related to the fact that only a spherical tumor shape was considered and that the analysis assumed a uniform distribution of microspheres and activity concentrations within the tumor and the normal liver compartments. Clearly, real patient tumors are not all spherical in shape. Additionally, the microsphere and activity concentrations are known to be heterogeneous on the sub-mm scale within the tumor and the normal liver compartments. While some anecdotal information has been gathered, there are no validated models for the expected heterogeneity of the microsphere distribution on the microscopic scale.

Although the reported results are for idealized and simplistic tumor shapes (ignoring the complex morphology of tumors) with uniform distributions (ignoring the heterogeneity of in vivo uptake distributions), the prescribed single-compartment doses were modeled on practical tumor characteristics such as the diameter and uptake. The trends and modulation of the prescribed dose reported are expected to serve as reliable guides for clinicians. We further acknowledge that the prescribed dose factor may have additional dependencies on parameters that were not addressed in this work, such as the number of particles used, the specific microsphere activity, or the vascular capacitance. Yet, the reported values could serve in a foundational paradigm as a starting point for the prescription of the practical single-compartment dosimetry commonly used for segmental and curative radioembolization treatments to help to improve the probability of achieving tumor CPN for a wide range of tumor sizes and uptakes.

The proposed ablative dosimetry model, based on specific tumor characteristics, can improve treatment efficacy by facilitating controlled prospective treatment planning that targets the dose according to the tumor margins in ablative radioembolization, rather than the ad hoc approaches typically employed, such as the application of 400 Gy to all radiation segmentectomies. Activity and dose modifications that focus on damaging tumors in clinical practice while maintaining sufficient liver function for untreated liver volumes have shown little adverse events, as evidenced by the use of radioembolization doses greater than 500–1000 Gy in the reported literature [7,28]. Furthermore, the proposed ablative dosimetry model is clinically practical because it is applicable to the routinely used single-compartment dosimetry models that are ubiquitous in ablative radioembolization settings [29].

## 5. Conclusions

This work described three dose metrics and their threshold values believed to be necessary to achieve CPN after ^90^Y-radioembolization based on numerical mm-scale dose modeling and the available clinical CPN evidence: the mean tumor dose ($D_{mean}$ ≥ $D_{mean}^{CPN}$ = 1053 Gy), the point dose for the tumor rim that ensures complete tumor dose coverage ($D_{rim}$ ≥ $D_{rim}^{CPN}$ = 860 Gy), and the point dose 2 mm outside the visible tumor margin that accounts for the dose for microscopic disease ($D_{2\mathrm{mm}}$ ≥ $D_{2\mathrm{mm}}^{CPN}$ = 561 Gy). The behavior of $D_{mean}$, $D_{rim}$, and $D_{2mm}$ were characterized as analytical functions of the tumor diameters and tumor-to-normal-liver uptake ratios. Most importantly, based on the commonly use *Standard* dosimetry model, the prescribed doses necessary to satisfy all of the CPN metrics ($D_{mean}^{CPN}$, $D_{rim}^{CPN}$, and $D_{2mm}^{CPN}$) were tabulated for a large range of patient-specific situations encountered in routine clinical practice in terms of tumor diameters (1–7 cm) and T:N  uptake ratios (2:1–5:1).

## Figures and Tables

**Table 1:** *Standard* segmental doses (in Gy) needed to satisfy $D_{mean}$ ≥ $D_{mean}^{CPN}$ = 1053 Gy for a range of tumor diameters and tumor-to-normal-liver uptake ratios.

$\mathrm{Tumor}\;\mathrm{Diameter}\;(d_{t})$	Tumor-to-Normal-Liver Uptake Ratio (T:N)			
	2:1	3:1	4:1	5:1
1 cm	684	506	402	333
2 cm	595	415	318	258
3 cm	570	391	297	240
4 cm	559	380	288	232
5 cm	552	374	283	227
6 cm	547	370	279	224
7 cm	544	367	277	222

**Table 2:** *Standard* segmental doses (in Gy) required to satisfy either $D_{rim}$ ≥ $D_{rim}^{CPN}$ = 860 Gy or $D_{2\mathrm{mm}}$ ≥ $D_{2\mathrm{mm}}^{CPN}$ = 561 Gy for a range of tumor-to-normal-liver uptake ratios.

Dose Metrics	Tumor-to-Normal-Liver Uptake Ratio (T:N)			
	2:1	3:1	4:1	5:1
$D_{rim}$ ≥ 860 Gy	566	400	309	252
$D_{2mm}$ ≥ 561 Gy	490	400	338	293

**Table 3:** *Standard* segmental doses (in Gy) required to satisfy $D_{mean}$ ≥ $D_{mean}^{CPN}$ = 1053 Gy, $D_{rim}$ ≥ $D_{rim}^{CPN}$ = 860 Gy, and $D_{2mm}$ ≥ $D_{2\mathrm{mm}}^{CPN}$ = 561 Gy for a range of tumor diameters and tumor-to-normal-liver uptake ratios. The limiting dose threshold denoted is by (*) for $D_{mean}^{CPN}$, (†) for $D_{rim}^{CPN}$, and (‡) for $D_{2\mathrm{mm}}^{CPN}$.

$\mathrm{Tumor}\;\mathrm{Diameter}\;(d_{t})$	Tumor-to-Normal-Liver Uptake Ratio (T:N)			
	2:1	3:1	4:1	5:1
1 cm	684 *	506 *	402 *	333 *
2 cm	595 *	415 *	338 ‡	293 ‡
3 cm	570 *	400 ‡		
4 cm	566 †			
5 cm				
6 cm				
7 cm				

## Data Availability

Supporting data can be provided upon reasonable request to the corresponding author.
